# A pilot study of oxidative pathways in MS fatigue: randomized trial of N‐acetyl cysteine

**DOI:** 10.1002/acn3.51325

**Published:** 2021-03-06

**Authors:** Kristen M. Krysko, Antje Bischof, Bardia Nourbakhsh, Roland G. Henry, Nisha Revirajan, Michael Manguinao, Khang Nguyen, Amit Akula, Yan Li, Emmanuelle Waubant

**Affiliations:** ^1^ UCSF Weill Institute for Neurosciences Department of Neurology University of California San Francisco San Francisco California USA; ^2^ Division of Neurology Department of Medicine St. Michael’s Hospital Li Ka Shing Knowledge Institute University of Toronto Toronto Ontario Canada; ^3^ Department of Neurology Johns Hopkins University Baltimore Maryland USA; ^4^ Department of Radiology and Biomedical Imaging University of California San Francisco San Francisco California USA

## Abstract

**Objective:**

To assess feasibility, tolerability, and safety of N‐acetyl cysteine (NAC) for fatigue in progressive MS. Secondary objectives evaluated changes in fatigue and oxidative pathway biomarkers on NAC versus placebo.

**Methods:**

Individuals with progressive MS with Modified Fatigue Impact Scale (MFIS) > t38 were randomized 2:1 to NAC 1250mg TID or placebo for 4 weeks. The primary outcome was tolerability and safety. The secondary outcome to evaluate efficacy was MFIS change from baseline to week 4 between groups. Exploratory biomarker outcomes included change in blood GSH/GSSG ratio (reduced‐to‐oxidized glutathione (GSH)) and in vivo relative GSH using 7T MR spectroscopy (MRS) between groups. Fisher exact test was used for categorical and rank sum for continuous outcomes.

**Results:**

Fifiteen were randomized (10 NAC, 5 placebo; mean age 56.1 years, 80% female, median EDSS 6.0). At least one adverse event (AE) occurred in 60% on NAC versus 80% on placebo (*p* = 0.75). There were two AEs attributed to NAC in one patient (abdominal pain and constipation), with 94% adherence to NAC. MFIS decreased in both groups at week 4, with the mean improvement of 11‐points on NAC versus 18‐points on placebo (*p* = 0.33). GSH/GSSG ratio decreased on placebo (−0.6) and NAC (−0.1) (*p* = 0.18). Change in GSH levels to total creatine in anterior and posterior cingulate cortex, insula, caudate, putamen, and thalamus did not differ between groups.

**Interpretation:**

NAC was well‐tolerated in progressive MS, although reduction in fatigue on NAC was similar to placebo. Antioxidant blood and MRS biomarkers were not significantly altered by NAC, which could be due to dose, route of administration, time of sample collection, short half‐life, or lack of effect.

**Registered:**

clinicaltrials.gov NCT02804594.

## Introduction

Fatigue is a disabling and common symptom of multiple sclerosis (MS) and involves a subjective lack of physical or mental energy with usual activities,^1^ with a negative impact on quality of life.^2^, ^3^ The underlying pathophysiology of fatigue in MS is poorly understood, with no approved treatments.^4^ Pathophysiologic processes that may contribute to primary fatigue in MS include CNS injury with regional atrophy,^5^, ^6^, ^7^ peripheral inflammation,^8^ cytokine changes,^9^ neuroendocrine abnormalities,^10^, ^11^ and neurotransmitter levels such as glutamate.^12^, ^13^

It is unknown whether oxidative stress contributes to fatigue. However, disequilibrium between oxidants and antioxidants could contribute to neurodegenerative processes,^14^, ^15^, ^16^ and lower brain glutathione (GSH), a major endogenous antioxidant, was observed in MS compared to healthy controls using MR spectroscopy (MRS).^15^, ^17^, ^18^ Additionally, clinical worsening was associated with greater decline in frontal GSH in progressive MS,^15^ suggesting oxidative stress may play a role in neurodegeneration in MS. The potential role of oxidative stress in neurodegeneration prompted this study, specifically evaluating oxidative stress in progressive MS. Reduced GSH on MRS has also been detected in chronic fatigue syndrome, with lower GSH associated with worse fatigue,^19^ but it remains unknown whether GSH level is associated with fatigue in MS. This prompted the current pilot study to assess the efficacy of an antioxidant for treating fatigue in progressive MS, and to evaluate biomarkers related to oxidative stress.

N‐acetyl cysteine (NAC) is an antioxidant, serving as a glutathione substitute, with direct scavenging of free radicals and restoration of neuronal GSH by providing a source of cysteine, which is a rate‐limiting substrate for GSH synthesis, thus increasing intracellular GSH.^20^ NAC is approved for acetaminophen‐induced hepatotoxicity,^21^ with adverse events associated with high NAC doses most commonly including gastrointestinal (GI) side effects and rash, as well as bronchospasm and tachycardia.^22^ Its use in MS is appealing since it is safe with neuroprotective properties.^23^, ^24^, ^25^, ^26^, ^27^, ^28^ A study of NAC combined with glatiramer acetate in seven persons with relapsing‐remitting MS (age range 28–46 years) appeared safe (6 reports of headache, 5 of GI symptoms, 1 of rash), but did not assess fatigue.^29^ While general safety data are available for NAC, there are no safety data in older populations of progressive MS who may be on disease‐modifying therapies (DMT) other than glatiramer acetate, and this is relevant as the future study of neuroprotective agents such as antioxidant therapies may target those with progressive MS.

We aimed to evaluate associations of oxidative pathways and fatigue in MS through a pilot randomized placebo‐controlled trial evaluating an antioxidant, NAC, for patients with progressive MS and fatigue. We aimed to evaluate the feasibility, tolerability, and safety of NAC compared to placebo over 4 weeks, as well as to obtain preliminary data on changes in fatigue and oxidative pathway biomarkers on NAC versus placebo. Given reported safety and tolerability in other populations, we hypothesized a similar safety profile in our study. We hypothesized that NAC may reduce level of fatigue. Biomarkers included the use of 7T MRS to evaluate changes in relative GSH in brain regions associated with fatigue,^5^, ^6^, ^7^ with the hypothesis that GSH would increase in response to NAC. We also evaluated the association between GSH and glutamate levels on MRS with the presence of fatigue at baseline. We hypothesized that fatigue would be associated with decreased GSH and glutamate, the latter supported by two studies demonstrating worsening MS fatigue with NMDA glutamate receptor antagonist treatment.^12^, ^30^

## Methods

### Trial design

This was a phase 2 randomized, placebo‐controlled, parallel‐group, double‐blind single‐center 4‐week pilot study of NAC compared to placebo on fatigue in progressive MS. Individuals who did not meet the criteria for fatigue as defined by the Modified Fatigue Impact Scale (MFIS) level at screening and who were otherwise eligible participated in a baseline visit and served in a nonfatigued MS control comparison group that did not receive the study drug.

### Participants

Participants were recruited from November 2016 to April 2018 through clinic and physician referrals from the University of California San Francisco (UCSF) Multiple Sclerosis Center. Individuals were eligible for the randomized portion of the study if they were 18–75 years in age, met 2010 McDonald criteria^31^ for MS of progressive^32^ subtype (primary or secondary progressive) with at least 1 year since symptom onset, Expanded Disability Status Scale (EDSS) score 2.0 to 6.5, and had self‐reported fatigue with MFIS > 38 at the screening visit. This EDSS range was required to capture individuals with at least some MS‐related disability and to allow measurement of all study outcomes, some of which required ambulation. If they otherwise met these criteria, but MFIS ≤ 38 at screening, they were eligible to participate as a nonfatigued control.

Exclusion criteria included MS relapses in the previous 3 months, steroid treatment within the prior month, pregnancy or breastfeeding, history of bleeding disorders, asthma requiring treatment, active gastrointestinal ulcers, secondary causes of fatigue, aspartate aminotransferase (AST) or alanine aminotransferase (ALT) > 2 times upper limit of normal, current treatment for active malignancy or metastatic malignancy treated in the past year, alcohol or substance use disorder, depression with Hospital Anxiety and Depression Scale (HADS) ≥ 15, allergy to NAC, or planned surgery or move within 10 weeks. Individuals were also excluded if receiving or about to start interferon‐beta or immunosuppressive medications as these can be associated with fatigue, or if starting or changing the dose of MS DMT within 3 months of baseline to avoid additional factors that could affect symptoms over the short study period. They were also excluded if on medications/supplements with glutamatergic or antioxidant properties or medications used for fatigue within 2 weeks of baseline, if starting or changing the dose of benzodiazepines, antidepressants, antipsychotics, antihistamines, or stimulants within a month of screening, or if on an anticoagulant.

The study was approved by the institutional review board at UCSF (16‐19826). All participants provided written informed consent. The trial was registered on clinicaltrials.gov (NCT02804594).

### Interventions

Individuals entering the randomized phase of the study received NAC 1250 mg (2 capsules of 625 mg each) or placebo three times per day (TID) for 4 weeks, taken with breakfast, lunch, and dinner. The total daily dose of 3750 mg per day was chosen since a similar dose of 35 mg/kg twice daily resulted in a detectable increase in CSF NAC concentration in Parkinson’s disease (PD).^24^ The placebo capsules had identical shape, size, and color to capsules containing NAC. Both NAC and placebo capsules were supplied by Wellspring Compounding Pharmacy (Berkeley, California). NAC is FDA‐approved for non‐neurologic indications, and FDA Investigational New Drug (IND) status was obtained for this study in progressive MS (IND number: 127814). Study medication bottles were collected at the end of the study to assess adherence. Those participating as nonfatigued MS controls did not receive any study interventions.

### Randomization and blinding

Eligible individuals were randomized 2:1 to NAC or placebo, with more assigned to NAC to improve recruitment and evaluate safety in a larger number of individuals compared to if equally allocated. A concealed randomized allocation schedule was generated by a team member not participating in the assessment of participants, and this was sent to the study pharmacy, which prepared and distributed blinded study drug to the UCSF research coordinator based on the randomization schedule. Patients, treating physicians, study personnel, and radiologists remained blinded to treatment assignment throughout the study.

### Outcomes

The primary outcome was feasibility, safety, and tolerability of NAC for 4 weeks, measured by differences in reported adverse events (AE) between NAC and placebo groups. AEs included the development or worsening of any undesirable symptom, sign, or medical condition occurring after starting the study drug and were graded according to the Common Terminology Criteria for Adverse Events (CTCAE). Serum creatinine, AST, and ALT were measured at screening and week 4. Expected AEs included GI side effects based on prior reports,^22^ although there was no specific protocol‐defined action in the setting of these.

The main secondary outcome to assess efficacy was change in fatigue as measured by MFIS at week 4 compared to baseline on NAC versus placebo. The MFIS is a 21‐item validated questionnaire evaluating fatigue over the last 28 days, including domains of physical (9 items), cognitive (10 items), and psychosocial (2 items) fatigue.^33^ The score ranges from 0 to 84, with higher scores indicating more severe fatigue, and has an estimated minimal clinically important difference of four points.^34^

Other secondary efficacy outcomes included the Fatigue Severity Scale (FSS) as another validated measure of fatigue. This includes nine questions on a 7‐point Likert scale, with the total score being the mean of item scores, with higher scores indicating more severe fatigue.^35^ Another secondary outcome included the validated Quality of Life in Neurological Disorders (Neuro‐QOL) fatigue item bank,^36^ with higher scores indicating worse fatigue‐related quality of life. Additional tertiary outcomes included the 9‐hole peg test (9‐HPT) and timed 25‐foot walk (T25FW), both timed, measured in duplicate and averaged.^37^ The Symbol Digits Modalities Test (SDMT) was administered to evaluate processing speed, with higher scores indicating faster‐processing speed.^38^

Exploratory outcomes included laboratory evaluation of oxidative stress and MRS measures as detailed below. Blood samples were collected at baseline for all participants and at week 4 for randomized participants, with whole blood mixed with 3% phosphoric acid. Samples were stored at −80°C and sent on dry ice to *Integrated Analytical Solutions,* where reduced GSH to oxidized glutathione (GSSG) ratio was measured.

Disability was measured at baseline with the EDSS score.^39^ Depression and anxiety at baseline were measured with the HADS.^40^

### Study procedures

There were three in‐person visits for randomized participants, including screening, baseline, and week 4 visits. There were two phone visits, at week 2 and week 6 (2 weeks after completing the last study drug dose). Tolerability of the study drug and AEs was assessed at week 2, 4, and 6. Clinical, laboratory, and MRS outcomes were measured at baseline and week 4. MFIS and FSS were re‐evaluated 2 weeks after discontinuation of the study drug by phone call (week 6). Visits were performed in the morning to maximize consistency of assessments and to allow measurement of the main outcomes (MFIS, FSS, labs, MRS) within 2 h of the morning study drug dose. Those not randomized but participating as nonfatigued MS controls participated in screening and baseline visits and had questionnaires, research laboratories, and MRS performed at baseline. Study data were entered and managed using REDCap (Research Electronic Data Capture).^41^, ^42^

### MR acquisitions

All MR scans were performed using a 32‐channel receive‐only array with a volume transmit head coil (NOVA Medical, Wilmington, MA, USA) on a GE 7T MR950 scanner (GE Healthcare, Waukesha, WI, USA). Anatomical imaging consisted of a sagittal scout (repetition time [TR]/echo time [TE] = 6/2 msec), T1‐weighted magnetization‐prepared rapid gradient echo method (MPRAGE) (TR/TE/inversion time [TI] = 4/2/1350 msec, matrix size = 256 × 256, field of view (FOV) = 256 × 256 mm^2^, 176 slices, voxel size = 1 × 1 × 1 mm^3^), T2‐weighted Cube‐FLAIR images (TR/TE/TI = 8000/140/2272 msec, matrix = 256 × 256, FOV = 256 × 256 mm^2^, 196 slices, voxel size = 1 × 1 × 1 mm^3^), and MR spectroscopy. Two MRS acquisitions were obtained in order to obtain relative glutathione and glutamate levels in the regions of interest (ROI). 3D MR spectroscopic imaging (MRSI) data were obtained parallel to the anterior commissure–posterior commissure line with full coverage of the thalamus (TE/TR = 20/2000 msec, matrix = 16–20 × 22 × 8, spatial resolution = 1 cm^3^, interleaved flyback echo‐planar trajectory applied in the anterior/posterior direction, total acquisition time ~10 min).^43^ GSH‐edited semi‐LASER^44^, ^45^ MR spectra were obtained using VAPOR water suppression^46^ and semi‐LASER localization^44^, ^45^ with the TE/TR being 72/3000ms. The editing pulses were placed at 4.5 ppm and 10 ppm in the two cycles. The voxels were located at the anterior cingulate cortex (ACC) and posterior cingulate cortex (PCC) with the voxel size being 25 × 25 × 25 and 30 × 20 × 20 mm^3^, respectively.

### MR postprocessing

First, brain extraction and N4 bias field correction^47^ were performed on the T1‐weighted images. Next, FAST segmentation algorithm^48^ was used to generate masks of gray matter, white matter, and cerebral spinal fluid.^49^ Segmentation of cortical (ACC, PCC, insula) and subcortical (caudate, putamen, thalamus) brain ROIs were performed using the Harvard–Oxford cortical and subcortical structural atlas.^50^ The segmented masks were then resampled to the orientation of the 3D MRSI data, and the percentage of each brain structure within each spectral voxel was calculated. Quality control involved manual review of images to exclude misplaced voxels and those containing MS lesions.

Postprocessing of the 3D MRSI datasets was performed using a previously published methodology.^43^, ^51^ Spectral arrays were processed with phase and frequency corrections individually for each coil and combined with weighting by coil sensitivities. For each ROI, spectral voxels that overlapped by at least 20% with the anatomical ROI (confirmed with manual location checking) and did not contain demyelinating lesions were averaged after phase and frequency corrections and then quantified using the LCModel^52^ using a simulated basis set. Only those voxels with relative Cramer–Rao lower bounds (CRLBs) <10 % for total creatine (t:Cr) and <20 % for glutamate and glutathione were included in the analysis.

The GSH‐edited semi‐LASER data were processed with phase and frequency corrections individually for each coil, and then combined with weighting by coil sensitivities. The difference spectra, the subtraction of edited from nonedited spectra, and nonedited spectra were quantified by LCModel^52^ using simulated basis sets. GSH with CRLB lower than 20% was included, and GSH:tCr was used in the analysis.

### Statistical analysis

#### Randomized participants

Those randomized to NAC and placebo were compared qualitatively at baseline to evaluate for balance on demographic and disease characteristics, as well as baseline fatigue, EDSS, HADS, and SDMT.

Safety and tolerability analyses included all individuals who received at least 1 dose of the study medication. The frequency of adverse events was compared between NAC and placebo groups with Fisher exact test. Moderate and serious adverse events were also compared between groups.

Analyses of efficacy outcomes were performed with intention‐to‐treat principles (excluding 1 participant randomized to placebo who withdrew consent prior to receiving study drug). For the main efficacy outcome, change in MFIS score from baseline to week 4 was compared between NAC and placebo groups with rank sum test. Similarly, secondary and tertiary outcomes including change in FSS, Neuro‐QOL fatigue item bank, 9‐HPT, T25FW, and SDMT, as well as change in physical, cognitive, and psychosocial components of the MFIS, from baseline to week 4 were compared between NAC and placebo groups with rank sum test. Change in MFIS and FSS from week 4 to 6 were also compared between NAC and placebo groups with rank sum test.

Analyses of exploratory MRS outcomes included analysis of change in GSH:tCr in ROIs including the insula and deep gray structures (caudate, putamen, thalamus) using 3D MRSI and ACC and PCC using GSH‐edited MRS from baseline to week 4 between NAC and placebo groups with rank sum test. GSH/GSSG ratio change from baseline to week 4 was also compared between NAC and placebo groups with rank sum test. Evaluation of whether change in GSH/GSSG ratio in blood was associated with change in GSH:tCr on MRS in the same ROIs over the 4‐week period was performed with Spearman correlation. Associations between change in total MFIS with GSH/GSSG ratio change and MRS GSH:tCr change were evaluated with Spearman correlation. Associations between the timing of the last study drug dose and research blood sampling and MRS with biomarkers were evaluated with Spearman correlation.

#### Baseline comparisons in fatigued versus nonfatigued groups

Baseline characteristics of the group categorized as fatigued (screening MFIS > 38) and nonfatigued (screening MFIS ≤ 38) were compared qualitatively at baseline on demographic and disease characteristics, as well as baseline fatigue, EDSS, and HADS.

Fatigue level as measured by MFIS at baseline was evaluated for association with GSH/GSSG ratio and MRS GSH:tCr and glutamate:tCr in the same ROIs as above with Spearman correlation. The GSH/GSSG ratio and MRS GSH:tCr and glutamate:tCr values were also compared between those with versus without fatigue using rank sum test. Glutamate:tCr on MRS was quantified with 3D MRSI for all ROIs.

This was a pilot trial, and no data were available to inform power calculations, so these were not performed, and the sample size was limited by available resources. Due to the exploratory nature of these analyses, we did not correct for multiple comparisons. All analyses were two‐sided, and alpha of 0.05 was used. Analyses were performed with STATA 15 (College Station, TX).

## Results

### Participants

**Figure 1:**
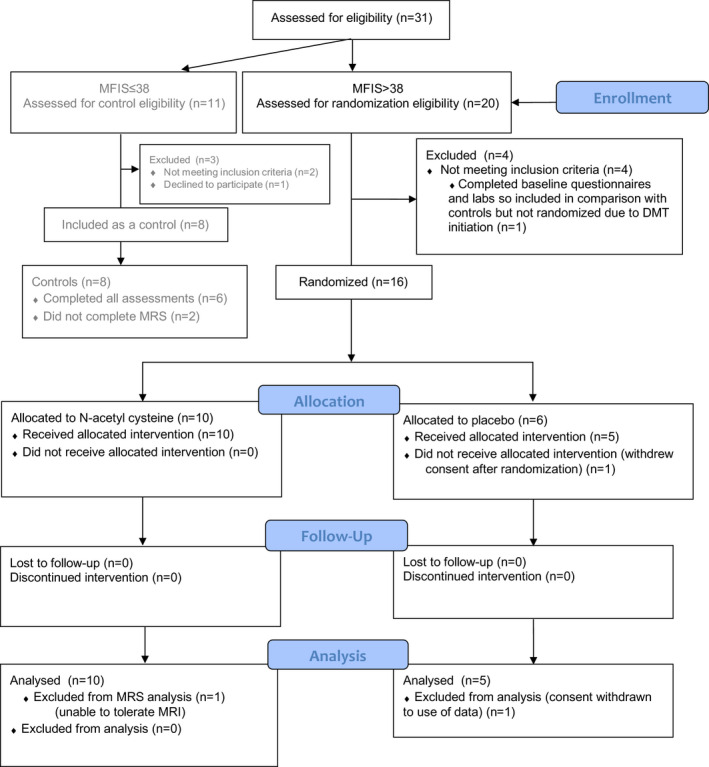
CONSORT diagram of participant enrollment. DMT disease‐modifying therapy; MFIS Modified Fatigue Impact Scale; MRS MR spectroscopy.

Thirty‐one individuals with MS were evaluated for eligibility from November 2016 to April 2018, and 20 had screening MFIS > 38. Four of these did not meet inclusion criteria for the randomized portion of the study, so 16 were randomized. One randomized to placebo did not receive the study intervention, and data were not analyzed as this participant withdrew consent, so five of six individuals allocated to placebo completed treatment and were analyzed. Ten individuals were allocated to NAC, and all completed follow‐up and were analyzed (Fig. 1). Nine on NAC and 5 on placebo completed MRS at baseline and week 4.

One additional participant with MFIS > 38 completed baseline questionnaires and laboratory tests, but was not eligible for randomization due to new DMT initiation, and was retained in comparison of fatigued and nonfatigued groups. Of 11 individuals with screening MFIS ≤ 38, eight were eligible and included as controls, of whom six completed MRS assessments. Thus, 16 fatigued and eight nonfatigued patients were compared at baseline.

### Randomized participants: NAC versus Placebo

Baseline characteristics and baseline measures of outcomes of those randomized to NAC (*n* = 10) and placebo (*n* = 5) were well‐balanced except age, which was higher in the placebo group (Tables 1 and 2).

**Table 1:** Baseline characteristics of participants.

Characteristic	All Fatigue (*n* = 16)^1^	NAC (*n* = 10)	Placebo (*n* = 5)	Nonfatigue MS (*n* = 8)
Age, mean years (SD)	**56.4 (10.5)**	51.3 (9.2)	65.7 (6.8)	**65.1 (6.7)**
Female sex, *n* (%)	**12 (75%)**	8 (80%)	4 (80%)	**5 (62.5%)**
Disease duration, median years (IQR)	**13.3 (6.9–19.8)**	14.9 (8.0–20.8)	12.3 (5.8–16.2)	**20.6 (13.6–26.9)**
White race, *n* (%)	**14 (87.5%)**	8 (80%)	5 (100%)	**7 (87.5%)**
Hispanic or Latino ethnicity, *n* (%)	**1 (6.3%)**	1 (10%)	0 (0%)	**0 (0%)**
MS subtype, *n* (%)				
Primary progressive	**11 (68.8%)**	6 (60%)	4 (80%)	**3 (37.5%)**
Secondary progressive	**5 (31.3%)**	4 (40%)	1 (20%)	**5 (62.5%)**
DMT use, *n* (%)	**10 (62.5%)**	7 (70%)	3 (60%)	**3 (37.5%)**
Ocrelizumab	**7 (43.8%)**	4 (40%)	3 (60%)	**2 (25%)**
Rituximab	**2 (12.5%)**	2 (20%)	0 (0%)	**0 (0%)**
Alemtuzumab	**1 (6.3%)**	1 (10%)	0 (0%)	**0 (0%)**
Dimethyl fumarate	**0 (0%)**	0 (0%)	0 (0%)	**1 (12.5%)**
Biotin use, *n* (%)	**4 (25%)**	3 (30%)	1 (20%)	**3 (37.5%)**
EDSS, median (IQR)	**6.0 (4.0–6.0)**	6.0 (4.0–6.0)	6.0 (3.5–6.0)	**5.0 (3.25–6.5)**
HADS depression, median (IQR)	**7 (5–8)**	7 (6–8)	7 (3–7)	**1.5 (1–2.5)**
HADS anxiety, median (IQR)	**6.5 (4–7)**	7 (4–7)	5 (5–6)	**1 (0–3.5)**

The bold columns of data demonstrate the fatigue versus nonfatigue MS comparisons.

DMT disease‐modifying therapy; EDSS Expanded Disability Status Scale; HADS Hospital Anxiety and Depression Scale; IQR interquartile range; NAC N‐acetyl cysteine; MS multiple sclerosis; SD standard deviation.

^1^ This includes patients randomized to NAC and placebo, and 1 additional patient with fatigue who was not randomized.

**Table 2:** Baseline outcome measures.

Characteristic	All Fatigue (*n* = 16)^1^	NAC (*n* = 10)	Placebo (*n* = 5)	Nonfatigue MS (*n* = 8)
MFIS, median (IQR)	**50 (45.5–56.5)**	48.5 (46–56)	50 (45–52)	**21.5 (14.5–35)**
FSS, median (IQR)	**5.7 (4.3–6.3)**	5.8 (5.0–6.2)	5.4 (3.6–5.6)	**2.9 (1.6–4.1)**
Neuro‐QOL fatigue, median (IQR)	**58 (53.5–64)**	59.5 (54–66)	53 (50–57)	**27.5 (23**–**35)**
9‐hole peg test (dominant), median (IQR)	**26.9 (21.5**–**34.7)**	29.3 (22.5–38.9)	25.3 (21.2–26.9)	**28.0 (23.5**–**33.0)**
9‐hole peg test (nondominant), median (IQR)	**31.4 (25.1**–**38.5)**	35.1 (28.8–40.1)	25.0 (23.9–26.6)	**29.0 (25.2**–**32.7)**
25‐foot walk, median seconds (IQR)	**6.8 (5.1**–**10.2)**	9.1 (5.6–10.2)	6.0 (5.1–6.8)	**9.9 (5.1**–**15.0)**
SDMT, median (IQR)	**39 (26.5**–**45)**	38 (29–43)	42 (42–47)	**42.5 (38.5**–**55.5)**
GSH:tCr ratio on MRS, median (IQR)				
ACC (by GSH‐edited MRS)^2^	**0.177 (0.147**–**0.208)**	0.187 (0.096–0.201)	0.151 (0.148–0.191)	**0.124 (0.098**–**0.254)** ^3^
PCC (by GSH‐edited MRS)^2^	**0.133 (0.106**–**0.167)**	0.130 (0.114–0.154)	0.167 (0.105–0.180)	**0.136 (0.111**–**0.149)** ^3^
Insula (by 3D MRSI)^2^	**0.378 (0.321**–**0.536)**	0.37 (0.324–0.402)	0.499 (0.374–0.578)	**0.317 (0.182**–**0.381)** ^3^
Caudate (by 3D MRSI)^2^	**0.381 (0.364**–**0.521)**	0.374 (0.262–0.900)	0.364 (0.058–0.521)	**0.272 (0.181**–**0.426)** ^3^
Putamen (by 3D MRSI)^2^	**0.384 (0.322**–**0.601)**	0.401 (0.322–0.419)	0.604 (0.209–0.674)	**0.260 (0.207**–**0.301)** ^3^
Thalamus (by 3D MRSI)^2^	**0.206 (0.183**–**0.302)**	0.281 (0.204–0.313)	0.192 (0.117–0.281)	**0.258 (0.223**–**0.537)** ^3^
GSH/GSSG blood ratio, median (IQR)	**2.37 (1.55**–**2.94)**	2.41 (1.48–3.31)	2.39 (1.87–2.42)	**2.54 (2.26**–**2.95)** ^4^

The bold columns of data demonstrate the fatigue versus nonfatigue MS comparisons.

ACC anterior cingulate cortex; FSS Fatigue Severity Scale; GSH glutathione; GSSG glutathione disulfide; IQR interquartile range; NAC N‐acetyl cysteine; MFIS Modified Fatigue Impact Scale; MRS MR spectroscopy; MS multiple sclerosis; PCC posterior cingulate cortex; QOL quality of life; SDMT Symbol Digit Modalities Test; tCr total creatine.

^1^ This includes patients randomized to NAC and placebo, and 1 additional patient with fatigue who was not randomized.

^2^ Sample size by brain region: ACC: NAC *n* = 6, placebo *n* = 4, fatigue *n* = 11, nonfatigue MS control *n* = 3; PCC: NAC *n* = 8, placebo *n* = 5, fatigue *n* = 14, nonfatigue MS control *n* = 5; Insula: NAC *n* = 6, placebo *n* = 4, fatigue *n* = 11, nonfatigue MS control *n* = 5; Caudate: NAC *n* = 3, placebo *n* = 3, fatigue *n* = 9, nonfatigue MS control *n* = 4; Putamen: NAC *n* = 6, placebo *n* = 3, fatigue *n* = 11, nonfatigue MS control *n* = 5; Thalamus: NAC *n* = 6, placebo *n* = 4, fatigue *n* = 12, nonfatigue MS control *n* = 4.

^3^ Fatigue versus nonfatigue comparison rank sum by brain region: ACC: *p* = 0.70; PCC: *p* = 0.71; Insula: *p* = 0.16; Caudate: *p* = 0.28; Putamen: *p* = 0.079; Thalamus: *p* = 0.43.

^4^ Fatigue versus nonfatigue comparison rank sum *p* = 0.46.

#### Safety and tolerability

There was no difference in the number of AEs between NAC and placebo groups (*p* = 0.75). Overall, there were 16.8 AEs per person‐year on NAC compared to 20.7 on placebo (Table 3). There were no serious AEs, and there was one moderate AE in each group. Most AEs were not attributed to the study drug. Only two AEs in one patient on NAC were attributed to the study drug and included abdominal pain and constipation, which both resolved upon dose interruption and did not recur with drug resumption, which occurred after 6 days. One patient on NAC had a headache. No participants suspended treatment due to an AE. Serum creatinine, AST, and ALT were within normal limits for all patients at week 4. Mean adherence was 97% on placebo and 94% on NAC, including doses missed due to a suggested dose interruption, to follow intention‐to‐treat principles.

**Table 3:** Adverse events in NAC and placebo groups.

	NAC (*n* = 10)	Placebo (*n* = 5)
Number of adverse events^1^	12	6
Number of adverse events per person‐year	16.8	20.7
Number of adverse events by patient, *n* (%)		
None	4 (40%)	1 (20%)
1	1 (10%)	2 (40%)
2	4 (40%)	2 (40%)
3	1 (10%)	0 (0%)
Number of moderate adverse events	1	1
Number of serious adverse events	0	0
Specific adverse events (*n*)	Abdominal pain (1)	Urinary tract infection (1)
	Constipation (1)	Increased fatigue (1)
	Common cold (1)	Gait disturbance (1)
	Sialadenitis (1)	Arthralgia (1)
	Muscle weakness (1)	Depression (1)
	Gait disturbance (1)	Back pain (1)
	Headache (1)	
	Anxiety (1)	
	Agitation (1)	
	Insomnia (1)	
	Injury to back (1)	
	Injury to face secondary to fall (1)	

NAC N‐acetyl cysteine.

^1^ Number of adverse events did not differ in NAC and placebo groups, with *p* = 0.75 for Fisher exact test for difference in number of adverse events between groups.

#### Efficacy

**Figure 2:**
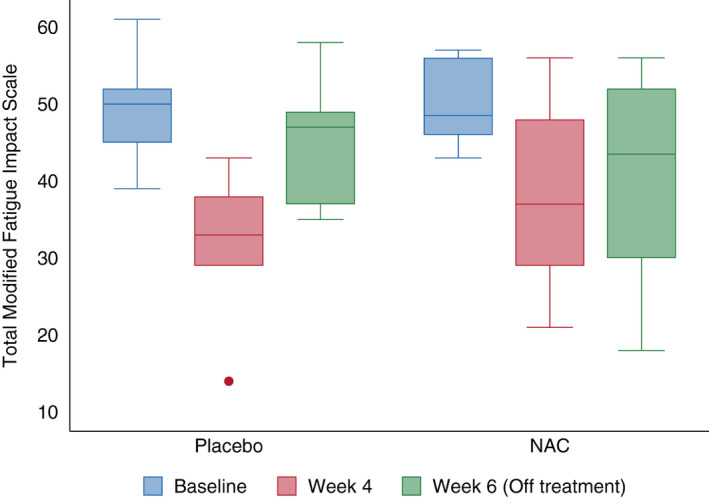
Modified Fatigue Impact Scale (MFIS) score at baseline, week 4 and week 6 in NAC and placebo groups. Higher MFIS indicates greater fatigue. MFIS score improved after 4 weeks on both NAC and placebo. At week 6, 2 weeks after stopping the study drug, there was more sustained improvement in fatigue on NAC than placebo, although this did not reach statistical significance. NAC N‐acetyl cysteine.

There was improvement in fatigue on both NAC and placebo, with an 11‐point improvement in MFIS on NAC (95% CI −22.0 to −0.8) and an 18‐point improvement on placebo (95% CI −37.2 to 1.2) (*p* = 0.33) (Table 4, Fig. 2). Both groups had improvements in the physical (*p* = 0.62), cognitive (*p* = 0.27), and psychosocial (*p* = 0.52) components of the MFIS with no statistically significant difference. Fatigue as measured by FSS (*p* = 0.76) and Neuro‐QOL (*p* = 0.50) also improved to a similar extent on NAC and placebo.

**Table 4:** Change in clinical, MRI, and laboratory measures in NAC and placebo groups.

	NAC (*n* = 10)	Placebo (*n* = 5)	*p* ^2^
Modified fatigue impact scale (MFIS)^1^			
MFIS mean change baseline to week 4	−11.4 (95% CI −22.0 to −0.8)	−18.0 (95% CI −37.2 to 1.2)	0.33
Physical MFIS change	−5.9 (95% CI −11.8 to 0.0)	−8.4 (95% CI −19.3 to 2.5)	0.62
Cognitive MFIS change	−4.4 (95% CI −9.4 to 0.6)	−7.8 (95% CI −15.0 to −0.6)	0.27
Psychosocial MFIS change	−1.1 (95% CI −2.3 to 0.1)	−1.8 (95% CI −3.8 to 0.2)	0.52
MFIS mean change week 4 to week 6	+1.8 (95% CI −8.1 to 11.7)	+13.8 (95% CI −5.2 to 32.8)	0.18
Physical MFIS change	+2.4 (95% CI −3.3 to 8.1)	+6.6 (95% CI 1.6 to 14.8)	0.22
Cognitive MFIS change	−0.3 (95% CI −4.1 to 3.5)	+5.6 (95% CI −5.0 to 16.2)	0.24
Psychosocial MFIS change	−0.4 (95% CI −1.2 to 0.4)	+1.6 (95% CI −0.7 to 3.9)	0.04
Fatigue severity scale (FSS)^1^			
FSS mean change baseline to week 4	−0.8 (95% CI −2.3 to 0.7)	−0.7 (95% CI −3.0 to 1.6)	0.76
FSS mean change week 4 to week 6	+0.6 (95% CI −0.5 to 1.7)	+0.8 (95% CI −0.4 to 2.0)	0.42
Neuro‐QOL fatigue item bank^1^			
Neuro‐QOL mean change baseline to week 4	−12.9 (95% CI −22.4 to −3.4)	−8.6 (95% CI −23.8 to 6.6)	0.50
GSH concentration on MRS			
GSH:tCr ratio median change baseline to week 4, IQR			
ACC (by GSH‐edited MRS)^3^	+0.029 (0.005–0.034)	−0.001 (−0.063–0.033)	0.39
PCC (by GSH‐edited MRS)^3^	+0.002 (−0.065–0.037)	0.000 (−0.029–0.008)	1.00
Insula (by 3D MRSI)^3^	+0.033 (−0.082–0.147)	−0.108 (−0.171–0.006)	0.20
Caudate (by 3D MRSI)^3^	−0.118 (−0.601– −0.094)	−0.082 (−0.201–0.311)	0.28
Putamen (by 3D MRSI)^3^	−0.067 (−0.177–0.000)	−0.101 (−0.312–0.146)	0.80
Thalamus (by 3D MRSI)^3^	+0.009 (−0.007–0.098)	+0.004 (−0.036−0.111)	1.00
GSH/GSSG ratio in blood			
GSH/GSSG ratio mean change baseline to week 4	−0.1 (95% CI −0.5 to 0.4)	−0.6 (95% CI −1.4 to 0.2)	0.18

ACC anterior cingulate cortex; CI confidence interval; FSS Fatigue Severity Scale; GSH glutathione; GSSG glutathione disulfide; IQR interquartile range; NAC N‐acetyl cysteine; MFIS Modified Fatigue Impact Scale; MRS MR spectroscopy; PCC posterior cingulate cortex; QOL quality of life; tCr total creatine.

^1^ Higher scores on the MFIS, FSS, and Neuro‐QOL indicate worse fatigue, so a negative change indicates improvement, whereas a positive change indicates worsening compared to prior.

^2^ Rank sum test comparing change between NAC and placebo groups.

^3^ Sample size by brain region: ACC, Insula, Thalamus: NAC *n* = 6, placebo *n* = 4; PCC: NAC *n* = 8, placebo *n* = 5; Caudate: NAC *n* = 3, placebo *n* = 3; Putamen: NAC *n* = 6, placebo *n* = 3.

From week 4 to 6, after being off the study drug for 2 weeks, fatigue worsened in both groups, but to a greater extent in the placebo (14‐point worsening) compared to NAC (2‐point worsening) group, although this did not reach statistical significance (*p* = 0.18). This pattern was noted across domains of fatigue, particularly the psychosocial MFIS domain, where the placebo group worsened after discontinuing the study drug, but the NAC group did not (*p* = 0.04). On the FSS, both NAC and placebo groups had increased fatigue after 2 weeks off the study drug to a similar extent (*p* = 0.42).

Other clinical measures, including 9‐hole peg test, 25‐foot walk speed, and SDMT, did not show a meaningful change over the 4‐week treatment period, with no difference between NAC and placebo groups (Supplementary Table).

#### Exploratory oxidative pathway biomarkers

**Figure 3:**
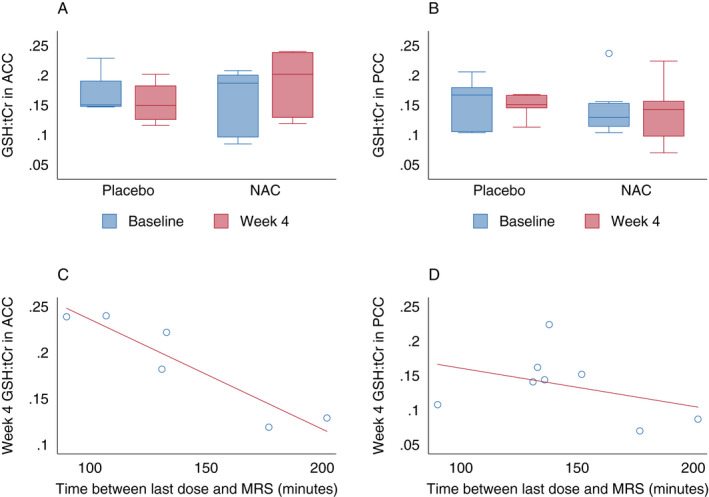
GSH‐edited MR spectroscopy in anterior and posterior cingulate cortex in NAC and placebo groups. GSH:tCr in placebo and NAC groups at baseline and week 4 in anterior cingulate cortex (ACC; A) and posterior cingulate cortex (PCC; B). Association between time of last study drug dose from MRS and GSH:tCr on MRS at week 4 in NAC group in ACC (C) and PCC (D). GSH glutathione; MRS MR spectroscopy; NAC N‐acetyl cysteine; tCR total creatine.

Exploratory evaluation of change in GSH:tCr from in vivo 7T MRS did not show a significant difference between NAC and placebo groups in the ACC (*p* = 0.39) or PCC (*p* = 1.00) by GSH‐edited MRS (Fig. 3), or in the insula (*p* = 0.20), caudate (*p* = 0.28), putamen (*p* = 0.80), or thalamus (*p* = 1.00) by 3D MRSI (Table 4). While the timing of MRS from the last study drug dose at week 4 did not differ between NAC and placebo groups, there was a median of 133 min (IQR 117–152) from the last dose to MRS. The time between the last dose of the study drug and MRS was strongly associated with GSH:tCr in the ACC in the NAC group (Spearman rho (*ρ*) −0.83, *p* = 0.042), with higher values in those with a shorter interval from the last NAC dose (Fig. 3), although this relationship was not as strong in other ROIs (PCC *ρ* −0.26, *p* = 0.53; insula *ρ* −0.26, *p* = 0.62; caudate *ρ* −0.50, *p* = 0.67; putamen *ρ* −0.39, *p* = 0.38), and there was no relationship in the thalamus (*ρ* 0.036, *p* = 0.94).

In blood, GSH/GSSG ratio declined in both groups over 4 weeks, with a slightly greater decline on placebo (−0.6) than NAC (−0.1), although this did not reach statistical significance (*p* = 0.18). Median time from the last dose of the study drug to blood sample at week 4 was 11 min (IQR 5–21) and did not differ between groups. The time from the dose to blood sample was not associated with GSH/GSSG ratio, although there was a narrow range in timing.

There was no significant consistent association between change in blood GSH/GSSG with change in GSH:tCr on MRS in the ROIs (Table 5). No significant association was found between change in MFIS and change in MRS GSH:tCr in the ROIs of interest on NAC (Table 5). In the placebo group, a decline in the blood GSH/GSSG ratio over 4 weeks was associated with increased fatigue (*ρ* −1.0, *p* < 0.001), whereas on NAC there was no strong relationship.

**Table 5:** Relationship between Modified Fatigue Impact Scale (MFIS), GSH:tCr on MRS, and GSH/GSSG ratio on blood at baseline, and changes in these measures over 4 weeks in NAC and placebo groups using Spearman correlation with *p*‐value in brackets.

	MRS GSH:tCr^1^, ^2^						Blood GSH/GSSG^1^, ^2^
	ACC^3^	PCC^3^	Insula^4^	Caudate^4^	Putamen^4^	Thalamus^4^	
Randomized (*n* = 10 NAC, *n* = 5 placebo)							
Change in MFIS^5^							
NAC	−0.20 (0.70)^6^	0.29 (0.49)^7^	0.03 (0.96)^6^	0.50 (0.67)^13^	0.14 (0.79)^6^	0.71 (0.11)^6^	0.21 (0.56)
Placebo	−0.20 (0.80)^8^	−0.50 (0.39)^9^	0.80 (0.20)^8^	−1.00 (<0.001)^13^	−0.50 (0.67)^13^	0.40 (0.60)^8^	−1.0 (<0.001)
Change in blood GSH/GSSG^2^								
NAC	0.43 (0.40)^6^	−0.52 (0.18)^7^	−0.20 (0.70)^6^	−1.00 (<0.001)^13^	−0.37 (0.47)^6^	0.43 (0.40)^6^	—
Placebo	0.20 (0.80)^8^	0.50 (0.39)^9^	−0.80 (0.20)^8^	1.00 (<0.001)^13^	0.50 (0.67)^13^	−0.40 (0.60)^8^	—
Baseline (*n* = 24)							
MFIS^5^	−0.013 (0.96)^10^	−0.11 (0.67)^11^	0.17 (0.53)^12^	0.27 (0.38)^14^	0.44 (0.084)^12^	−0.17 (0.53)^12^	−0.20 (0.34)

ACC anterior cingulate cortex; GSH glutathione; GSSG glutathione disulfide; NAC N‐acetyl cysteine; MFIS Modified Fatigue Impact Scale; MRS MR spectroscopy; PCC posterior cingulate cortex; tCr total creatine.

^1^ For rows of data from randomized portion of the study, these reflect change values between week 4 and baseline, whereas measured values were compared at baseline.

^2^ Higher values of GSH:tCr and GSH/GSSG indicates higher GSH, an antioxidant. Higher values of change in these ratios indicate an increase in GSH over the 4 weeks.

^3^ Measured by GSH‐edited MR spectroscopy.

^4^ Measured by 3D MRSI.

^5^ Higher MFIS indicates a greater fatigue level. Higher values of change in MFIS indicate an increased level of fatigue over the 4 weeks.

^6^ Available data in *n* = 6.

^7^ Available data in *n* = 8.

^8^ Available data in *n* = 4.

^9^ Available data in *n* = 5.

^10^ Available data in *n* = 14.

^11^ Available data in *n* = 19.

^12^ Available data in *n* = 16.

^13^ Available data in *n* = 3.

^14^ Avialable data in *n* = 13.

### Baseline comparisons in fatigued versus nonfatigued

Compared to patients with progressive MS without significant fatigue, those with fatigue in this study were younger with shorter disease duration, more likely to have primary progressive MS, and more likely to be on DMT. The fatigued group had a similar level of disability, but higher levels of anxiety and depression as measured by the HADS (Tables 1 and 2).

The level of GSH:tCr from in vivo MRS in the ACC, PCC, insula, caudate, putamen, and thalamus, as well as the blood GSH/GSSG ratio were similar in the fatigued and nonfatigued groups (*p* > 0.05) (Table 2). There were also no strong relationships between the baseline fatigue level as measured by MFIS and GSH:tCr in the ROIs, or with GSH/GSSG in the blood (Table 5). Similarly, glutamate:tCr in the same ROIs with 3D MRSI did not differ between the fatigued and nonfatigued groups (data not shown), and was not associated with baseline MFIS (ACC *ρ* −0.43, *p* = 0.082; insula *ρ* −0.13, *p* = 0.64; caudate *ρ* −0.14, *p* = 0.64; putamen *ρ* −0.01, *p* = 0.97; thalamus *ρ* −0.13, *p* = 0.63), except in the PCC in which higher MFIS was associated with lower glutamate (*ρ* −0.48, *p* = 0.032).

## Discussion

In this pilot randomized, blinded placebo‐controlled trial of NAC for fatigue in progressive MS, NAC was well‐tolerated and appeared safe. There were occasional gastrointestinal side effects and headaches, as previously reported in MS and other populations.^22^, ^24^, ^29^, ^53^ Adherence to NAC was high over 4 weeks despite TID dosing, further supporting tolerability and feasibility. Improvement in fatigue over 4 weeks was clinically meaningful,^34^ but did not differ significantly between NAC and placebo groups. Interestingly, there appeared to be less rebound in the psychosocial domain of fatigue at the end of the treatment period in the NAC than placebo group, although this was not seen for the overall MFIS. GSH level on MRS was not changed by NAC and did not differ between those with and without fatigue. While the hypothesis that NAC was safe and tolerable was met, hypotheses of a beneficial effect of NAC on fatigue or MRS GSH biomarkers of oxidative stress were not met. The hypothesis of a difference in GSH between fatigued and nonfatigued groups was also not met.

At the oral NAC dose of 1250 mg TID, there was no significant increase in GSH in cortical or deep gray matter structures studied on MRS or blood GSH/GSSG ratio at week 4 compared to baseline, without a difference in change in these values from the placebo group. This is in contrast to a study in PD and Gaucher disease, in which a higher dose of intravenous NAC (150 mg/kg over 1 hour = 10,500 mg for a 70 kg individual) led to increased GSH in the occipital cortex on MRS, maximally at 90–110 min from the start of infusion, and increased blood GSH/GSSG, maximally at 60–75 min from the start of the infusion.^25^ A study of oral NAC in PD showed a detectable increase in CSF NAC levels 90 min after 35 mg/kg (2450 mg for a 70 kg individual) and 70 mg/kg doses, although that study did not evaluate for biological effects of NAC on intracellular GSH.^24^ Oral administration of a lower NAC dose (~18 mg/kg per dose for a 70 kg individual) and the longer time to MRS acquisition in our study (median 133 min), as well as the blood sampling at a median of only 11 min after NAC dosing, may have led to lack of detection of a biological effect of NAC on GSH concentration. Blood samples may have been collected too soon after a dose since peak NAC concentration occurs 1–2 h from an oral dose.^54^ Poor oral absorption, short half‐life, low dose, and timing of measurements all could have contributed to a lack of biological effect of NAC on GSH on MRS and blood measures in this study.

Without the hypothesized biological effect of NAC on GSH, it is not surprising that we did not detect an effect of NAC on fatigue. It remains unknown whether a higher dose of NAC may affect neuronal GSH level or fatigue. However, a recently published study of 24 individuals with MS randomized to NAC or standard of care, also did not detect an effect of NAC on fatigue.^53^ On the other hand, their treatment with intravenous 50 mg/kg NAC weekly with oral NAC 500 mg twice daily other days was associated with improved cerebral glucose metabolism in several brain areas compared to controls, as well as improved cognition and attention,^53^ although this study was unblinded without a placebo, making it difficult to draw conclusions.

In patients with progressive MS with and without substantial fatigue, we did not find differences in GSH ratio on MRS or GSH/GSSG ratio in the blood. While GSH has been reported to be reduced in MS compared to healthy controls,^15^, ^17^, ^18^ it did not appear to be associated with fatigue level in this study. However, the small sample size with a limited range of fatigue severity precludes definitive conclusions on the contribution of oxidative stress to fatigue in progressive MS. Glutamate:tCr ratio on MRS also did not differ between those with and without fatigue, and higher fatigue severity was only associated with decreased glutamate ratio in the PCC. It is possible that low glutamate concentrations may be associated with fatigue, as two studies demonstrated worsening of MS fatigue with an NMDA glutamate receptor antagonist (memantine).^12^, ^30^ Further studies are required to assess the association of low glutamate concentrations with fatigue as we observed this association in the PCC, but not in other brain regions.

This study demonstrates the feasibility of using 7T MRS to measure relative GSH levels in gray matter structures of individuals with progressive MS. This adds to the growing literature of the use of MRS to measure glutathione as a marker of oxidative stress in MS.^15^, ^17^, ^18^ Further longitudinal evaluation and validation of gray matter GSH measured by 7T MRS as a marker of oxidative stress in progressive MS would be valuable, with the potential of this measure to serve as a biomarker in future trials of neuroprotective agents targeting oxidative stress.

Limitations of this study include the small sample size given the pilot nature of the study. This led to a baseline imbalance between NAC and placebo groups in age, which could confound findings. Another limitation is the lack of evaluation of different doses, frequencies, or routes of administration of NAC, since a lack of effect of oral NAC 1250 mg TID on antioxidant biomarkers in this study could be due to dose and/or route of administration. In our study, GSH:tCr concentrations were higher in the ACC in those with MRS performed closer in time to the NAC dose, suggesting a potential effect that might be detectable with the acquisition of MRS at peak NAC serum levels. However, the optimal dose, frequency, and route of administration to maintain steady NAC serum levels, and thereby continuous antioxidant effects, has yet to be determined. Furthermore, the reproducibility of blood and MRS measurements was not evaluated, introducing potential bias from measurement error.

Further study is needed to better understand oral dosing and frequency of NAC that could achieve an effect on neuronal GSH before pursuing a larger study evaluating the effect on clinical markers of disease progression or fatigue in MS. A dose‐finding study of different doses and frequencies of NAC and associated effects on MRS GSH could be a useful next step. This study also highlights the strong placebo effect in evaluating treatment for fatigue, with substantial and clinically meaningful improvements in fatigue in both NAC and placebo groups. This can make it difficult to detect a treatment‐specific effect of a medication or behavioral intervention for fatigue, although strategies to harness the placebo effect for clinical treatment may prove useful if an ethical balance can be achieved.^55^

Overall, while NAC was safe and well‐tolerated, we did not find a benefit of oral NAC 1250 mg TID on fatigue in individuals with progressive MS. In this small study, we also did not find an association between markers of oxidative stress and fatigue in progressive MS. Further larger studies should explore whether oxidative stress contributes to primary fatigue or neurodegeneration in MS to determine whether this could be targeted for the treatment of this disabling and common symptom or neurodegenerative aspects of the disease.

## Conflicts of Interest

Kristen Krysko was funded by a Sylvia Lawry Physician Fellowship through the National Multiple Sclerosis Society (FP‐1605‐08753 (Krysko)). She also had fellowship funding through Biogen. Antje Bischof reports no disclosures. Bardia Nourbakhsh reports personal fees from Jazz Pharmaceutical and grants from Genentech, outside the submitted work. Roland G Henry reports grants and personal fees from Roche/Genentech, personal fees from Novartis, personal fees from Genzyme, personal fees from Atara, personal fees from Celgene, outside the submitted work. Nisha Revirajan reports no disclosures. Michael Manguinao reports grants from Race to Erase MS, during the conduct of the study. Khang Nguyen reports no disclosures. Amit Akula reports no disclosures. Yan Li reports no disclosures. Emmanuelle Waubant reports personal fees from DBV, Jazz Pharmaceuticals, Emerald, outside the submitted work.

## Authors’ Contributions

KMK participated in the design of the study and analysis; conducted statistical analysis; acquired data; drafted the manuscript. AB participated in the design and performance of MRI analyses; revised the manuscript for intellectual content. BN participated in the conception and design of the study; revised the manuscript for intellectual content. RGH participated in the conception and design of the study; revised the manuscript for intellectual content. NR participated in the design of the study; major role in acquisition of data; revised the manuscript for intellectual content. MM participated in the design of the study; major role in acquisition of data; revised the manuscript for intellectual content. KN participated in the design and performance of MRI processing; revised the manuscript for intellectual content. AA participated in the design and performance of MRI processing; revised the manuscript for intellectual content. YL had a major role in the design and performance of MRI acquisition/processing/analyses; revised the manuscript for intellectual content. EW had a major role in the conception and design of the study; major role in acquisition of data; revised the manuscript for intellectual content.

## Supporting information


**Table S1**. Change in other clinical measures in NAC and placebo groups.
