# Supplementary material for: A pilot study of oxidative pathways in MS fatigue: randomized trial of N‐acetyl cysteine
Source: Ann Clin Transl Neurol. 2021 Mar 6;8(4):811–24. doi: 10.1002/acn3.51325 (PMC8045913; doi:10.1002/acn3.51325)
Supplement: Supplementary file 1 — Table S1. Change in other clinical measures in NAC and placebo groups. [file ACN3-8-811-s001.docx]

**Supplementary Table. Change in other clinical measures in NAC and placebo groups.**

|  | **NAC (n=10)** | **Placebo (n=5)** | ***p^a^*** |
| --- | --- | --- | --- |
| **9-hole peg test (dominant) mean change baseline to week 4^b^** | -3.3 (95% CI -9.5 to 2.8) | +1.1 (95% CI -2.9 to 5.0) | 0.16 |
| **9-hole peg test (non-dominant) mean change baseline to week 4^b^** | +1.1 (95% CI -2.9 to 5.0) | -1.1 (95% CI -2.5 to 0.2) | 0.57 |
| **25-foot walk mean change baseline to week 4,^b^ seconds** | +0.4 (95% CI -0.5 to 1.2) | +0.0 (95% CI -0.6 to 0.7) | 0.46 |
| **SDMT mean change baseline to week 4^c^** | -0.4 (95% CI -3.2 to 2.4) | +0.8 (95% CI -8.1 to 9.7) | 1.0 |

^a^Rank sum test comparing change between NAC and placebo groups. ^b^Higher scores on these measures indicate worse performance, so a negative change indicates improvement while a positive change indicates worsening compared to prior. ^c^Higher SDMT indicates better performance, so a positive change indicates improvement while a negative change indicates worsening compared to prior.

CI confidence interval; NAC N-acetyl cysteine; SDMT Symbol Digit Modalities Test.
